# Supplementary material for: Integrated Analysis of the Lung Microbiome and Metabolome Reveals Associations Between Amino Acid Metabolism and Pulmonary Fibrosis in a Bleomycin-Induced Mouse Model
Source: Int J Mol Sci. 2026 Jun 30;27(13):5895. doi: 10.3390/ijms27135895 (PMC13362081; doi:10.3390/ijms27135895)
Supplement: Supplementary file 1 [file ijms-27-05895-s001.zip › result/5.MetKeggEnrichment/B_28d.vs.C_28d/B_28d.vs.C_28d_all_KEGG_map/KEGG.map.html]

Pathway Enrichment


  

# The most enriched pathway terms of B\_28d.vs.C\_28d\_all

Statistic method: hypergeometric test

FDR correction method: Benjamini and Hochberg

| MapID | MapTitle | Pvalue | AdjustedPv | x | y | n | N | EnrichDirect | GeneIDs |
| --- | --- | --- | --- | --- | --- | --- | --- | --- | --- |
| map00830 | Retinol metabolism | 0.0234367762853173 | 0.0775074587427328 | 1 | 2 | 3 | 255 | Over | Com\_698\_pos |
| map00981 | Insect hormone biosynthesis | 0.0350165773570329 | 0.0775074587427328 | 1 | 3 | 3 | 255 | Over | Com\_527\_pos |
| map00900 | Terpenoid backbone biosynthesis | 0.0465044752456397 | 0.0775074587427328 | 1 | 4 | 3 | 255 | Over | Com\_527\_pos |
| map00270 | Cysteine and methionine metabolism | 0.0915443579317756 | 0.114430447414719 | 1 | 8 | 3 | 255 | Over | Com\_719\_pos |
